# Effects of Virtual Reality Motor-Cognitive Training for Older People With Cognitive Frailty: Multicentered Randomized Controlled Trial

**DOI:** 10.2196/57809

**Published:** 2024-09-11

**Authors:** Rick Yiu Cho Kwan, Justina Liu, Olive Suk Kan Sin, Kenneth N K Fong, Jing Qin, Joe Chi Yin Wong, Claudia Lai

**Affiliations:** 1 School of Nursing Tung Wah College Hong Kong China (Hong Kong); 2 School of Nursing The Hong Kong Polytechnic University Hong Kong China (Hong Kong); 3 Research Institute for Smart Ageing The Hong Kong Polytechnic University Hong Kong China (Hong Kong); 4 Board of Director Pok Oi Hospital Hong Kong China (Hong Kong); 5 Department of Rehabilitation Sciences The Hong Kong Polytechnic University Hong Kong China (Hong Kong); 6 Sengital Limited Hong Kong China (Hong Kong)

**Keywords:** virtual reality, motor-cognitive training, cognitive frailty, gamification

## Abstract

**Background:**

Cognitive frailty refers to a clinical syndrome in which physical frailty and mild cognitive impairment coexist. Motor-cognitive training and virtual reality (VR) have been used to launch various therapeutic modalities to promote health in older people. The literature advocates that motor-cognitive training and VR are effective in promoting the cognitive and physical function of older people. However, the effects on older people with cognitive frailty are unclear.

**Objective:**

This study examined the effects of VR motor-cognitive training (VRMCT) on global cognitive function, physical frailty, walking speed, visual short-term memory, inhibition of cognitive interference, and executive function in older people with cognitive frailty.

**Methods:**

This study used a multicentered, assessor-blinded, 2-parallel-group randomized controlled trial design. Participants were recruited face-to-face in 8 older adult community centers. Eligible participants were aged ≥60 years, were community dwelling, lived with cognitive frailty, had no dementia, and were not mobility restricted. In the intervention group, participants received VRMCT led by interventionists with 16 one-hour training sessions delivered twice per week for 8 weeks. In the control group, participants received the usual care provided by the older adult community centers that the investigators did not interfere with. The primary outcome was global cognitive function. The secondary outcomes included physical frailty, walking speed, verbal short-term memory, inhibition of cognitive interference, and executive function. Data were collected at baseline (T0) and the week after the intervention (T1). Generalized estimating equations were used to examine the group, time, and interaction (time × group) effects on the outcomes.

**Results:**

In total, 293 eligible participants enrolled in the study. The mean age of the participants was 74.5 (SD 6.8) years. Most participants were female (229/293, 78.2%), had completed primary education (152/293, 52.1%), were married (167/293, 57.2%), lived with friends (127/293, 43.3%), and had no VR experience (232/293, 79.5%). In the intervention group, 81.6% (119/146) of participants attended >80% (13/16, 81%) of the total number of sessions. A negligible number of participants experienced VR sickness symptoms (1/146, 0.7% to 5/146, 3%). VRMCT was effective in promoting global cognitive function (interaction effect: *P*=.03), marginally promoting executive function (interaction effect: *P*=.07), and reducing frailty (interaction effect: *P*=.03). The effects were not statistically significant on other outcomes.

**Conclusions:**

VRMCT is effective in promoting cognitive functions and reducing physical frailty and is well tolerated and accepted by older people with cognitive frailty, as evidenced by its high attendance rate and negligible VR sickness symptoms. Further studies should examine the efficacy of the intervention components (eg, VR vs non-VR or dual task vs single task) on health outcomes, the effect of using technology on intervention adherence, and the long-term effects of the intervention on older people with cognitive frailty at the level of daily living.

**Trial Registration:**

ClinicalTrials.gov NCT04730817; https://clinicaltrials.gov/study/NCT04730817

## Introduction

### Background

Cognitive frailty (also known as potentially reversible cognitive frailty) refers to a clinical syndrome in which physical frailty (including physical prefrailty or physical frailty) and mild cognitive impairment (MCI) coexist [1,2]. The prevalence in older people is increasing, as shown in a systematic review that showed that the pooled estimates of cognitive frailty prevalence in community-dwelling older populations increased from 6% (2012-2017) to 11% (2018-2020) [3]. Another systematic review showed that cognitive frailty is commonly seen in older people aged ≥60 years and that older age and a lower level of engagement in activities are associated with a higher risk of cognitive frailty [4]. It was found to be a significant predictor of adverse health outcomes, including all-cause mortality and dementia. Cognitive frailty was found to be a better predictor of adverse health outcomes (eg, dementia and depression) than physical frailty alone [5,6]. Recent evidence has shown that effective interventions enhancing cognitive and physical functions in older people with cognitive frailty are mostly multidimensional but commonly include the component of physical and cognitive training [2,7,8].

Motor-cognitive training (also known as dual-task training) refers to training in which both physical and cognitive training are performed at the same time (ie, thinking about how to solve a problem while moving) [9]. The Guided Plasticity Facilitation framework proposed that the combination of physical and cognitive activities has positive synergistic effects that exceed the positive effects of either cognitive or physical training alone because the facilitation effects of physical training and the guidance effects of cognitive training trigger a neurophysiological mechanism to promote neuroplasticity, as evidenced by the enhanced release of neurotrophic factors (eg, brain-derived neurotrophic factor) [10]. In the literature, evidence has shown that motor-cognitive training is effective in treating many neurological diseases (eg, chronic stroke and Parkinson disease) to improve gait performances and risk of falls [11,12], but its effects on cognitive function are not clear in people with Parkinson disease [13]. Recently, motor-cognitive training has been used to treat people with MCI, and there is preliminary evidence suggesting that it might improve cognitive function in addition to physical function [14-16].

Virtual reality (VR) refers to the use of a computer system that aims to immerse users in a virtual environment by replacing visual and aural environments to achieve a sense of presence so that users perceive themselves as being part of this virtual environment [17]. VR is often used as a platform to launch various therapeutic modalities in older people to promote various aspects of their health, including cognitive, physical, and mental health, with promising effects when compared with traditional training methods (eg, conducted by a therapist in real time) [18-20]. People with MCI commonly display impaired attentional processing and working memory capacity [21]. Physical activity adherence is proven to be poor in older people with cognitive impairment [22]. VR is an ideal platform to launch therapies because it allows for a more controllable intervention environment through simulation and it attracts participants’ attention, resulting in more interaction with the training contributing to better training results [23].

### Objectives

The literature advocates that the elements of motor-cognitive training and VR are effective in promoting the cognitive and physical functions of older people with various chronic conditions (eg, stroke and MCI) [11,14,24]. However, their effects on older people with cognitive frailty are unclear. A pilot study was conducted by our team in Hong Kong that examined the effects of a VR motor-cognitive training (VRMCT) system (ie, the same training system tested in this study) on 17 older people with cognitive frailty [25]. The preliminary evidence (ie, the within-group effect of 9 participants) of the pilot study supported that the VRMCT was effective in promoting cognitive and physical functions and reducing physical frailty and the intervention was feasible to be implemented in people with cognitive frailty [25]. Nevertheless, there are no full-powered randomized controlled trials to provide proof of its effects. Therefore, the objectives of this study were to examine the effects of VRMCT on global cognitive function, physical frailty, walking speed, visual short-term memory, inhibition of cognitive interference, and executive function in older people with cognitive frailty.

## Methods

### Trial Design

This study used a multicentered, assessor-blinded, 2-parallel-group, 1:1-allocation-ratio randomized controlled trial design. The CONSORT (Consolidated Standards of Reporting Trials) 2010 guidelines were followed to report on this trial [26]. This trial was registered at ClinicalTrials.gov with the last updated version on August 27, 2021 (ClinicalTrials.gov ID: NCT04730817).

### Participants

#### Overview

Participant recruitment was conducted in 8 older adult community centers (ie, District Elderly Community Centres), which provide support services at the district level in Hong Kong to enable older people to remain in the community and lead a healthy, respectful, and dignified life [27]. The research team produced a set of intervention promotional materials (eg, flyers, videos, newsletters, and presentation slides). The staff members of the older adult community centers used promotional materials with the institutional affiliation’s name displayed to invite interested and potentially eligible center members to enroll in this study. Upon the referral by the staff members of the older adult community centers and with the approval of the potential participants, the research team members conducted eligibility assessments and, subsequently, the demographic and outcome assessment for eligible participants at the older adult community centers. All eligible older people were invited to participate in this study.

#### Inclusion Criteria

The inclusion criteria were as follows:

1. The participants were aged ≥60 years.
2. The participants were community dwelling, defined as not living in a long-term care facility in the previous 12 months.
3. The participants had cognitive frailty, defined as the coexistence of MCI and physical frailty without concurrent dementia [2,28]. MCI is measured through a Montreal Cognitive Assessment (MoCA) score of <26/30 [29]. Physical frailty is measured as a Fried frailty phenotype (FFP) score of >0 [30]. The measurement of each phenotypic criterion followed the Fried method using local normative data [31]. Exclusion of possible dementia was measured through a MoCA score of <19/30 [32].

#### Exclusion Criteria

The exclusion criteria were as follows:

1. There was a confirmed diagnosis of dementia, defined as the documented diagnosis on the participants’ medical record.
2. The participants had restricted mobility, defined as a Modified Functional Ambulation Classification score of <7 [33].

### Interventions

#### Design

There were 2 parallel groups in this study. The intervention group received VRMCT, whereas the control group received usual care.

#### Intervention Group: VRMCT

**Figure 1:**
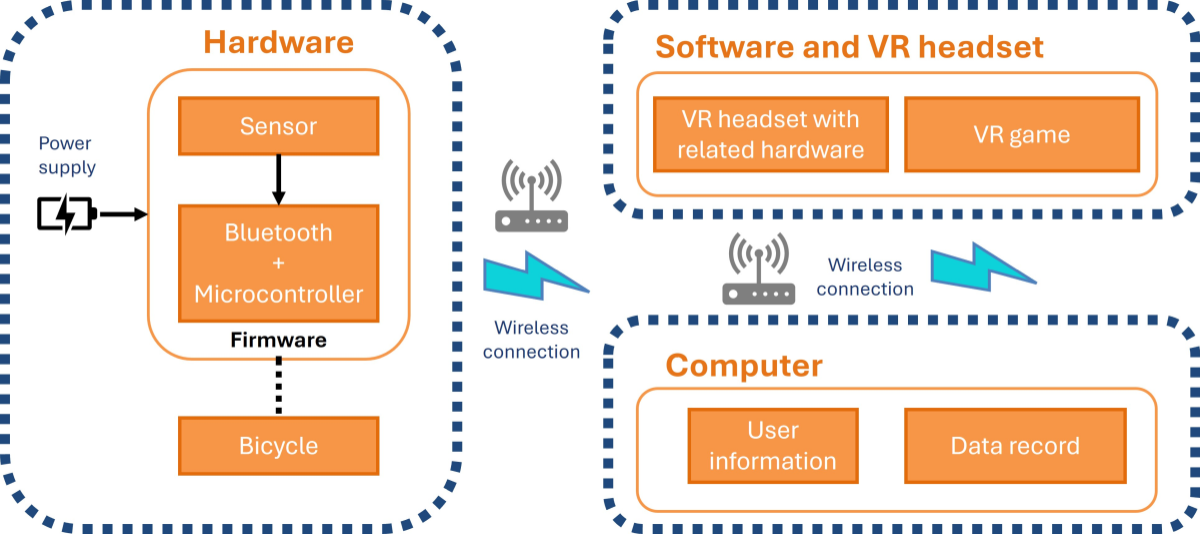
The design of the virtual reality (VR) motor-cognitive training system.

The intervention was to deliver physical and cognitive training simultaneously on the VR platform. As shown in Figure 1, the VRMCT system comprises 3 parts: hardware, software and VR headset, and computer. The hardware consists of a motion sensor, a microcontroller unit with a Bluetooth Low Energy feature, and a power-controlled integrated circuit. The hardware also consists of a bicycle, which is an underdesk ergometer with adjustable cycling resistance (ie, DeskCycle 2). The motion sensor is attached to the paddle of the bicycle to capture the cycling motion of the participants. Once the sensor captures motion data, it communicates with the microcontroller unit. The motion sensor is specifically designed for seamless integration with the system. The second part of the VRMCT system is the software and VR headset, which consists of a VR headset with related hardware, including a head-mounted VR display with a pair of headphones and wireless handheld controllers (ie, HTC Vive Focus Plus). The VR games are installed in the HTC Vive Focus Plus headset. Using the communication protocol, the motion sensor is connected wirelessly to the VR headset. As a result, the participants can control their movement in the virtual environment by cycling on the motion sensor–connected bicycle. The third part of the VRMCT system is the computer, which stores users’ information and records the data logged by the system during the process of training. Using a similar communication protocol, the computer is connected wirelessly to the software and VR headset to record the data.

**Figure 2:**
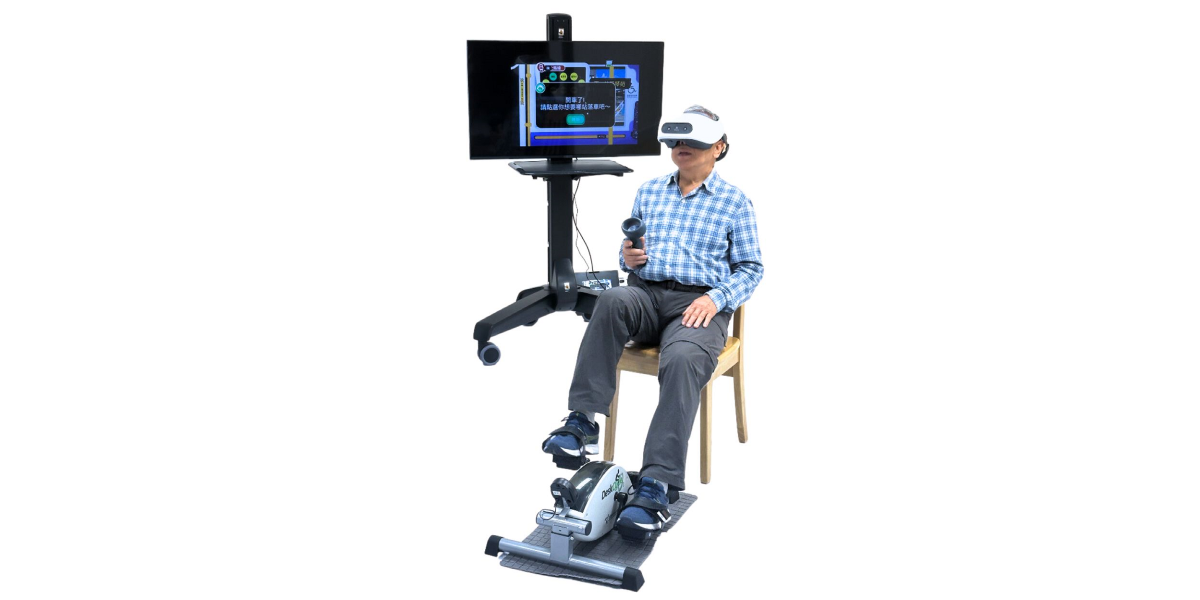
The setup of the virtual reality motor-cognitive training system.

The VR game was developed by an interprofessional team with the needed expertise, including nurses (RYCK, JL, and CL), an occupational therapist (KNKF), a social worker (OSKS), and computer engineers (JQ and JCYW) aiming to develop a prototype effective at promoting the health of older people with cognitive frailty and technically usable and accepted by them. As shown in Table 1, the VR game contains 8 different themes: orientation, finding a bus stop, reporting lost items, finding a supermarket, grocery shopping, cooking, finding a travel hot spot, and bird watching. They mimic and gamify the problems faced and activities undertaken by older people regularly. In week 1, orientation sessions were provided to the participants aiming to teach them to master all the commands needed in the training (eg, cycling the ergometer to control the movement and speed in the virtual world and using the handheld controller to select and unselect items). In the remaining weeks, participants were instructed to navigate the virtual world by cycling on the motion sensor–connected ergometer and solve the gamified problems by exercising their cognitive capabilities in the virtual world. For example, in week 2, participants were expected to navigate the virtual world to find a designated bus stop to take a bus to a supermarket to continue the game. Participants were expected to memorize and recognize the visual cues in the virtual environment (eg, traffic lights and building names) to find the designated bus stop; while they were doing so, they would be exercising their visuospatial abilities, as shown in Multimedia Appendix 1. As another example, in week 5, participants were expected to shop for food in the supermarket. Participants were expected to exercise their memory to recall the names of the food items needed for cooking, pay attention to find the image of the food items needed, and do the calculation to settle the payment, as shown in Multimedia Appendix 2. The illustration of the setup of the intervention on the participants is shown in Figure 2.

**Table 1:** Themes of the virtual reality motor-cognitive training system.

Week	Themes	Purposes	Cognitive functions required
1	Orientation	Learn all the commands in the training	—^a^
2	Finding a bus stop	Find a bus stop on a given route in a city	Attention Visuospatial function
3	Reporting lost items	Report a lost item found on the street to police	Problem-solving Visuospatial function
4	Finding a supermarket	Find a particular supermarket in the city	Attention Visuospatial function
5	Grocery shopping	Shop in a supermarket for a list of food items and calculate the price of the selected items	Memory Attention Calculation
6	Cooking	Flip eggs at a specific time interval	Mental processing speed
7	Finding a travel hot spot	Find a travel hot spot in a park	Attention Visuospatial function
8	Bird watching	Identify the birds in a park that were shown at the beginning of the game	Attention Memory

^a^Not applicable.

The VR motor-cognitive intervention was provided in 8 older adult community centers in Hong Kong. The duration of the whole intervention was 8 consecutive weeks, including two 1-hour sessions each week. The actual use of VR devices was limited to 30 minutes per session to minimize possible cyber fatigue or cybersickness because a longer exposure time increases the risk of VR sickness [34]. The duration and frequency were designed according to the pilot study, which was shown to be sufficient to yield clinical outcomes on global cognitive function and physical frailty [25]. Within the same week, the theme of the 2 training sessions was the same, but the levels of difficulty of the 2 sessions in terms of cognitive challenge increased. Participants attended the easier one first, followed by the harder one. The total number of training sessions was 16, and each training session lasted for 1 hour, including briefing, setup, VRMCT (30 minutes), and posttraining observation. In the briefing, participants were primed with the training objectives of the session (eg, to find a bus stop) and reminded about the essential controls needed. In the setup, all items of the VRMCT system (ie, motion sensor–connected ergometer, VR headset, and the computer) were wirelessly connected via Bluetooth. The resistance level of the ergometer was then set, with a goal of progressive increase to the maximum tolerable level according to the participants’ previous training experience (eg, knee pain, muscle ache, and fatigue) and motivation to increase the training dose (ie, the resistance level of the ergometer). After that, VR headset fitting was conducted on the participants aiming to make sure that the position of the VR headset allowed for the best-quality 3D vision experienced by the participants. In the VRMCT, the participants followed the computer-generated and interventionist’s instructions to complete the training tasks. Each session was expected to be completed within 20 to 30 minutes according to participants’ cognitive performance. The session ended if the training lapsed for 30 minutes. Finally, immediately after the training, participants were placed in a safe sitting position with the VR headset removed and observed for at least 10 minutes. This was because potential adverse effects were commonly observed in older people (eg, VR sickness, dizziness, and eyestrain) during the period [35]. Participants were only allowed to leave if they had no adverse symptoms.

During the intervention, the participants wore the head-mounted VR display and were immersed in the virtual world of the self-developed VR game with the voice-over and written instructions in the VR game and the verbal instructions of the accompanying on-site interventionists. The interventionists were undergraduate nursing students who were trained by following an implementation protocol (eg, setup of the system, technical problem-solving, common adverse effect identification, and provision of cues to participants in given situations) to guide participants through the training sessions. The intervention was delivered by the interventionists at a 1:1 interventionist-to-participant ratio. The interventionists delivered the interventions and accompanied the participants throughout the whole intervention period. All interventionists completed a standardized training, including a 1-hour training and returned demonstration. The training contents included the setup of the equipment, communication with older people with cognitive frailty, equipment fitting on participants, training objectives of each session, instructions provided to participants, troubleshooting, observable adverse effects, and emergency management. To ensure intervention fidelity, a research team member (author RYCK) regularly and randomly site visited the performance of the interventionists’ implementation. A checklist mapped with the intervention implementation protocol was used to guide the observation of the interventionists. When deviations from the intervention implementation protocol were observed, remedial training was provided to the interventionists by the research team member (RYCK) at the site. When technical problems were observed or reported, they were fixed by the technical team (JCYW).

To ensure adherence to the intervention, the participants were reminded by either the interventionists or the older adult community center staff members to attend the training 1 day before the training. All participants who completed the training for >80% of the sessions (ie, 13/16, 81%) were presented with a certificate of completion in a mini ceremony of completion held in the older adult community center. Some scheduled sessions could not be attended by participants because of some unforeseeable reasons (eg, illnesses) and were rescheduled to ensure that 16 sessions were provided to all participants. To ensure an adequate intervention dose, up to 2 makeup sessions were arranged within 2 weeks after the completion of the scheduled period of 8 consecutive weeks for participants who missed some sessions.

Participants accessed the intervention at the designated older adult community centers. The intervention was delivered by the interventionists who were authorized to operate the VRMCT system. Only authorized interventionists could deliver the intervention and operate the system. The systems were owned by the research team. All eligible participants in the study received the intervention for free.

Apart from the intervention provided by the research team, participants led their normal lives and were not restricted from enrolling in or engaging in any activities (eg, physical or social activities) provided by the community centers or organizations outside the community centers.

#### Waitlist Control Group: Usual Care

Participants in this group received usual care. The research team did not provide any interventions to the participants in this group during the 8-week intervention period. Similarly to the participants in the intervention group, participants in the control group were also not restricted from enrolling on or engaging in any activities (eg, physical or social activities) provided by the community centers or organizations outside the community centers. However, a waitlist control method was adopted, and an 8-week and 16-session VRMCT was provided to all participants in this group after the completion of follow-up data collection (ie, 8 weeks after receiving usual care from the baseline).

### Outcomes

#### Overview

Demographic data were collected at baseline (ie, T0 at week 0) that included age, number of chronic illnesses according to the Charlson Comorbidity Index [36], BMI, gender, educational level, marital status, living status, and VR experience. Outcome variables included global cognitive function (primary), physical frailty, walking speed, visual short-term memory, inhibition of cognitive interference, and executive function. These were measured at both baseline (ie, T0 at week 0) and the week immediately after the completion of the intervention (ie, T1 at week 9).

#### Global Cognitive Function

Global cognitive function was measured using the MoCA, which comprises 30 dichotomous items [32]. One point is assigned to one correct answer. The total score ranges from 0 to 30. A higher score indicates a higher level of cognitive function. The MoCA has a strong correlation with the Mini-Mental State Examination (*r*=0.90) and Saint Louis University Mental Status Examination (*r*=0.83) [37], as well as a good criterion validity in detecting MCI [29,32].

#### Physical Frailty

Physical frailty was measured using the FFP, which assesses physical frailty based on 5 components: weight loss, exhaustion, low physical activity, slow gait, and weakness [30]. One point is assigned to the presence of one component, and the FFP score ranges from 0 to 5. A higher score indicates a higher level of frailty.

#### Walking Speed

Walking speed was measured using the Timed Up and Go test, which assesses the total time needed for a participant to stand up from a chair, walk a 3-meter distance, walk back to the chair, and sit down. A longer time needed indicates a slower walking speed [38].

#### Verbal Short-Term Memory

Verbal short-term memory was measured using the digit span test [39]. The digit span test is conducted through forward and backward digit recall and scored using the longest forward and backward digit string recalled without error. A higher score indicates a higher level of verbal short-term memory. The digit span test was validated to classify people with different levels of memory impairment or disorder [40,41]. Verbal short-term memory was assessed as an outcome because verbal short-term memory training was integrated as part of the VRMCT intervention.

#### Inhibition of Cognitive Interference

Inhibition of cognitive interference was measured using the Stroop Color and Word Test. This popular test assesses a person’s ability to inhibit cognitive interference when processing a specific stimulus while a second stimulus is simultaneously introduced. This outcome was assessed because participants were also trained to respond to 2 stimuli (cognitive and motor) as part of the VRMCT intervention. The number of correct answers produced in a fixed time (ie, 30 seconds) were counted separately in the color test, word test, and color-word test. The inhibition score is calculated using the formula *I* = color-word test – ([word test + color test] / 2) [42]. A higher score indicates a higher level of inhibition of cognitive interference.

#### Executive Function

Executive function was measured using the Trail Making Test (TMT). Time is counted for the completion of both the TMT part A and TMT part B. The TMT score is calculated by dividing the TMT part B by the TMT part A [43]. A higher score indicates a lower level of executive function. This outcome was assessed because participants were also trained to exercise their executive function as part of the VRMCT intervention.

### Ancillary Variables

Ancillary variables included VR sickness, unstructured untoward effects on participants, and unplanned events in the training sessions. These were measured during the intervention period every time after the completion of each session of the training.

VR sickness was measured using the Virtual Reality Sickness Questionnaire (VRSQ) [44]. The VRSQ consists of 9 simulator sickness symptoms: general discomfort, fatigue, eyestrain, difficulty focusing, headache, fullness of head, blurred vision, dizziness, and vertigo. The severity of each symptom is rated using a 4-point Likert scale (ie, 0=*never*; 3=*very*). The total score was the summation of all item scores and was then converted to a percentage score. A higher score indicates a higher severity of VR sickness. The VRSQ was validated as a reliable tool (Cronbach α=0.85-0.89) [44].

Open-ended questions were posed to all participants (ie, “Do you have any uncomfortable symptoms apart from the symptoms covered in the VRSQ?”) and interventionists (“Did you observe any unusual symptoms on the participants or conditions throughout the intervention period?”). The questions were asked through a questionnaire launched on a web-based platform (ie, Qualtrics [Qualtrics International Inc]).

### Sample Size

We adopted a previous power analysis using the web-based software GLIMMPSE and used a general linear mixed model [45]. We set the level of significance at 0.05, the power at 0.9, the number of repeated measures at 2 (ie, T0 and T1), the number of groups at 2 (ie, the intervention and control groups), and the allocation ratio between the 2 groups at 1:1. To estimate the effects, we referred to the interaction (ie, group × time) effect on global cognitive function (ie, primary outcome) observed in the pilot study with a highly identical design [25]. The estimated sample size was 220. We assumed a dropout rate of 6%, as observed in the pilot study [25]. The total sample size was expected to be at least 234 participants, with 117 in each group.

### Randomization

A permuted block randomization with a block size of 8 in an intervention-to-control ratio of 1:1 was used. A list of random numbers of either 1 or 2 (ie, 1=intervention; 2=control) of each block was generated independently by author RYCK using the RAND formula in Microsoft Excel (Microsoft Corp). After each round of eligibility screening, a list of unique participant codes was assigned to each eligible participant by the participant recruitment team. A research assistant assigned all eligible participants to either the intervention or control group by matching the participant codes with the list of random group numbers. To ensure concealment, the research assistant managing the random group allocation did not participate in any part of recruitment.

### Blinding

All outcome assessors were blinded to the group labels. We also endeavored to blind the participants to the group label at baseline assessment through an arrangement in which the random group allocation was only conducted after the completion of the baseline assessment. However, it was not possible to blind the interventionists or participants given the nature of the study.

### Statistical Methods

SPSS (version 26; IBM Corp) was used to conduct the data analysis. The demographic and outcome variables at continuous levels of measurement were first checked for normality, which is assumed when the absolute skewness is <2 and the absolute kurtosis is <7 [46]. Continuous variables with normality assumed were described using mean and SD or otherwise using median and IQR. Variables at categorical levels of measurement were described using frequency and percentage. To test the effects of the intervention over the 6 outcomes, 6 independent generalized estimating equations were used. The independent variables included group (ie, intervention vs control), time (ie, T0 vs T1), and group × time. The dependent variables were the 6 outcomes of the study (ie, cognitive function, physical frailty, walking speed, inhibition of cognitive interference, executive function, and walking speed). Missing values were estimated using the generalized estimating equations [47]. The level of significance was set at 0.05. Intention-to-treat analysis was adopted.

### Ethical Considerations

All participants signed the written informed consent form. Before signing the written consent form, the participants were well informed about the expected benefits of the intervention, their rights (eg, right of withdrawal), the possibility of being assigned to either intervention or control, and possible risks. All data are anonymized to ensure confidentiality. Because all participants received the interventions (waitlist control was used), no compensation was provided to participants for their participation in the research. The ethical clearance for research involving human subjects for this study was provided by the institutional review board of the Hong Kong Polytechnic University (reference HSEAR202100115001-01).

## Results

### Participant Flow

**Figure 3:**
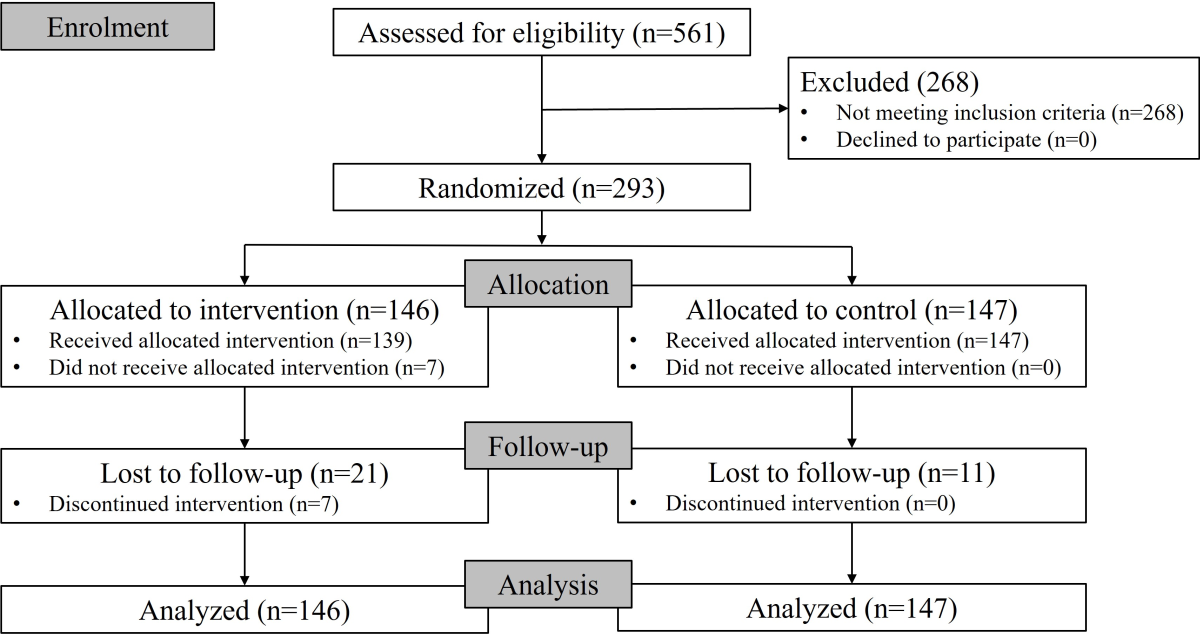
Participant flowchart.

This study was conducted from January 2021 to April 2023. As shown in Figure 3, a total of 561 participants were assessed for eligibility. In total, 47.8% (268/561) of the participants were excluded because they did not fulfill the eligibility criteria. No eligible participants declined to participate. A total of 293 eligible participants were randomly allocated to the 2 groups: 146 (49.8%) were allocated to the intervention group, and 147 (50.2%) were allocated to the control group. In the intervention group, 4.8% (7/146) of the participants did not receive the allocated intervention, defined as attending 0 training sessions. A total of 81.6% (119/146) of the participants attended ≥81% (13/16) of the expected sessions, 5.9% of the participants attended 50% (8/16) to 75% (12/16) of the expected sessions, and 12.5% of the participants attended 0% to 44% (7/16) of the expected sessions. In the control group, all 147 participants received usual care. After the completion of the intervention, in the intervention group, 14.4% (21/146) of the participants were lost to follow-up, and 23% (7/21) of these participants discontinued the intervention, defined as attending 0 training sessions. In the control group, 7.5% (11/147) of the participants were lost to follow-up.

### Baseline Data

As shown in Table 2, the mean age of the participants was 74.5 (SD 6.8) years, and most of the participants had no chronic illnesses (175/293, 59.7%). The mean BMI of the participants was 24.3 (SD 3.6) kg/m^2^. Most of the participants were female (229/293, 78.2%), had completed primary education (152/293, 52.1%), were married (167/293, 57.2%), lived with friends (127/293, 43.3%), and had no VR experience (232/293, 79.5%). The mean MoCA score was 21.3 (SD 2.4), the mean FFP score was 1.6 (SD 0.8), the median Timed Up and Go test score was 10.9 (IQR 3.6) seconds, the mean digit span forward test score was 7.1 (SD 1.2), the mean digit span backward test score was 3.8 (SD 1.3), the mean Stroop Color and Word Test score was –21.4 (SD 9.1), and the mean TMT score was 1.7 (SD 0.7).

**Table 2:** Demographic and outcome variables at baseline (N=293).

Variables				All		Intervention (n=146)		Control (n=147)
**Demographic variables**								
	Age (y), mean (SD)		74.5 (6.8)		75.2 (7.1)		73.9 (6.6)	
	**Number of CIs^a^, n (%)**							
		0	175 (59.7)		87 (59.6)		88 (59.9)	
		1-3	113 (38.6)		57 (39.0)		56 (38.1)	
		4-6	5 (1.7)		2 (1.4)		3 (2)	
	BMI (kg/m^2^), mean (SD)		24.3 (3.6)		24.4 (3.7)		24.2 (3.5)	
	**Gender, n (%)**							
		Men	64 (21.8)		36 (24.7)		28 (19)	
		Women	229 (78.2)		110 (75.3)		119 (81)	
	**Educational level, n (%)**							
		Tertiary	20 (6.8)		12 (8.2)		8 (5.4)	
		Secondary	98 (33.4)		44 (30.1)		54 (36.7)	
		Primary	152 (51.8)		81 (55.5)		71 (48.3)	
		No education	22 (7.5)		8 (5.5)		14 (9.5)	
	**Marital status, n (%)**							
		Unmarried	12 (4.1)		5 (3.4)		7 (4.8)	
		Married	167 (57.2)		90 (62.1)		77 (52.4)	
		Separated	2 (0.7)		0 (0)		2 (1.4)	
		Divorced	17 (5.8)		10 (6.9)		7 (4.8)	
		Widowed	9.4 (32.2)		40 (27.6)		54 (36.7)	
	**Living status, n (%)**							
		Living alone	101 (34.5)		49 (33.6)		52 (35.4)	
		Living with family	42 (14.3)		23 (15.8)		19 (12.9)	
		Living with friends	127 (43.3)		60 (40.1)		67 (45.6)	
		Living with others	22 (7.4)		14 (9.6)		8 (5.4)	
	**VR^b^ experience, n (%)**							
		Yes	60 (20.5)		18 (12.4)		42 (28.6)	
		No	232 (79.5)		127 (87.6)		105 (71.4)	
**Outcome variables**								
	MoCA^c^ score, mean (SD)		21.3 (2.4)		21.1 (2.42)		21.5 (2.4)	
	FFP^d^ score, mean (SD)		1.6 (0.8)		1.6 (0.8)		1.7 (0.8)	
	TUG^e^ score (seconds), median (IQR)		10.9 (3.6)		10.7 (3.1)		11.5 (4.1)	
	**VSTM^f^, mean (SD)**							
		DSF^g^ score	7.1 (1.2)		7.1 (1.3)		7.1 (1.1)	
		DSB^h^ score	3.8 (1.3)		3.8 (1.3)		3.8 (1.3)	
	SCWT^i^ score, mean (SD)		–21.4 (9.1)		–22.1 (9.4)		–20.8 (8.7)	
	TMT^j^ score, mean (SD)		1.7 (0.7)		1.7 (0.1)		1.6 (0.1)	

^a^CI: chronic illness.

^b^VR: virtual reality.

^c^MoCA: Montreal Cognitive Assessment.

^d^FFP: Fried frailty phenotype.

^e^TUG: Timed Up and Go test.

^f^VSTM: visual short-term memory.

^g^DSF: digit span forward test.

^h^DSB: digit span backward test.

^i^SCWT: Stroop Color and Word Test.

^j^TMT: Trail Making Test.

### Numbers Analyzed

As shown in Table 3, the number of participants with missing data on all variables in the intervention group, including both demographic and outcome variables, ranged from 0% to 28.8% (42/146) at baseline (T0) and from 14.4% (21/146) to 30.8% (45/146) at follow-up (T1). The number of participants with missing data for all variables in the control group ranged from 0% to 22.4% (33/147) at baseline (T0) and from 7.5% (11/147) to 27.9% (41/147) at follow-up (T1). The data for all participants who entered the randomized group allocation were analyzed (ie, n=146 in the intervention group and n=147 in the control group).

**Table 3:** The number of participants with missing data.

Variables		Intervention (n=146), n (%)		Control (n=147), n (%)	
		T0^a^	T1^b^	T0	T1
**Demographic variables**					
	Age (y)	2 (1.4)	—^c^	0 (0)	—
	Number of CIs^d^	1 (0.7)	—	0 (0)	—
	BMI	1 (0.7)	—	1 (0.7)	—
	Gender	0 (0)	—	0 (0)	—
	Educational level	1 (0.7)	—	0 (0)	—
	Marital status	1 (0.7)	—	0 (0)	—
	Living status	1 (0.7)	—	1 (0.7)	—
	VR^e^ experience	1 (0.7)	—	0 (0)	—
**Outcome variables**					
	MoCA^f^ score	1 (0.7)	21 (14.4)	0 (0)	11 (7.5)
	FFP^g^ score	1 (0.7)	21 (14.4)	0 (0)	11 (7.5)
	TUG^h^ score	1 (0.7)	21 (14.4)	0 (0)	14 (9.5)
	DSF^i^ score	1 (0.7)	21 (14.4)	1 (0.7)	13 (8.8)
	DSB^j^ score	1 (0.7)	21 (14.4)	1 (0.7)	14 (9.5)
	SCWT^k^ score	42 (28.8)	45 (30.8)	33 (22.4)	41 (27.9)
	TMT^l^ score	1 (0.7)	21 (14.4)	1 (0.7)	12 (8.2)

^a^T0: baseline.

^b^T1: follow-up.

^c^Demographic data were only collected at baseline.

^d^CI: chronic illness.

^e^VR: virtual reality.

^f^MoCA: Montreal Cognitive Assessment.

^g^FFP: Fried frailty phenotype.

^h^TUG: Timed Up and Go test.

^i^DSF: digit span forward test.

^j^DSB: digit span backward test.

^k^SCWT: Stroop Color and Word Test.

^l^TMT: Trail Making Test.

### Outcomes and Estimation

Cognitive function improved from T0 (mean MoCA score 21.12, SE 0.204) to T1 (mean MoCA score 22.10, SE 0.273) and reached the level of significance (within-group *P*<.001) in the intervention group but not in the control group (within-group *P*=.33). The extent of improvement in the intervention group was also significantly larger than that in the control group (group × time *P*=.03), as shown in Table 4.

**Table 4:** Outcome estimates.

Outcome and group		Values, mean (SE)			MD^a^ (95% CI)		*P* value			
		T0^b^	T1^c^			Within group		Between groups	Time	Group × time
**Global cognitive function (MoCA^d^)**										
	Intervention	21.12 (0.204)	22.10 (0.273)	0.97 (1.38 to 0.56)		<.001^e^		.89	<.001^e^	.03^e^
	Control	21.29 (0.200)	21.54 (0.299)	0.25 (0.73 to –0.23)		.33		—^f^	—	—
**Physical frailty (FFP^g^)**										
	Intervention	1.60 (0.060)	1.29 (0.068)	–0.31 (–0.17 to –0.46)		<.001^e^		.006^e^	.003^e^	.01^e^
	Control	1.69 (0.064)	1.66 (0.093)	–0.03 (0.15 to –0.21)		.74		—	—	—
**Walking speed (TUG^h^)**										
	Intervention	11.25 (0.311)	10.77 (0.242)	–0.48 (0.06 to –1.03)		.08		.02^e^	.78	.13
	Control	12.10 (0.454)	12.80 (0.829)	0.71 (2.13 to –0.72)		.33		—	—	—
**VSTM^i^: DSF^j^**										
	Intervention	7.13 (0.107)	7.43 (0.112)	0.30 (0.51 to 0.08)		.006^e^		.44	.001^e^	.46
	Control	7.08 (0.096)	7.27 (0.108)	0.19 (0.38 to –0.01)		.04^e^		—	—	—
**VSTM: DSB^k^**										
	Intervention	3.88 (0.104)	3.92 (0.149)	0.04 (0.34 to –0.27)		.82		.80	.48	.74
	Control	3.82 (0.109)	3.92 (0.115)	0.10 (0.33 to –0.13)		.40		—	—	—
**ICI^l^ (SCWT^m^)**										
	Intervention	–22.19 (0.897)	–21.33 (0.953)	0.86 (2.59 to –0.88)		.33		.34	.78	.31
	Control	–20.45 (0.814)	–20.94 (0.981)	–0.49 (1.41 to –2.39)		.62		—	—	—
**Executive function (TMT^n^)**										
	Intervention	1.68 (0.054)	1.51 (0.048)	–0.17 (–0.04 to –0.29)		.01^e^		.47	.12	.07
	Control	1.63 (0.056)	1.65 (0.063)	0.01 (0.16 to –0.13)		.85		—	—	—

^a^MD: mean difference.

^b^T0: baseline.

^c^T1: follow-up.

^d^MoCA: Montreal Cognitive Assessment.

^e^*P*<.05.

^f^Not applicable.

^g^FFP: Fried frailty phenotype.

^h^TUG: Timed Up and Go test.

^i^VSTM: visual short-term memory.

^j^DSF: digit span forward test.

^k^DSB: digit span backward test.

^l^ICI: inhibition of cognitive interference.

^m^SCWT: Stroop Color and Word Test.

^n^TMT: Trail Making Test.

Physical frailty decreased from T0 (mean FFP score 1.60, SE 0.060) to T1 (mean FFP score 1.29, SE 0.068) and reached the level of significance (within-group *P*<.001) in the intervention group but not in the control group (within-group *P*=.74), and the extent of reduction in the intervention group was also significantly larger than that in the control group (group × time *P*=.01), as shown in Table 4.

Executive function improved from T0 (mean TMT score 1.68, SE 0.054) to T1 (mean TMT score 1.51, SE 0.048) and reached the level of significance (within-group *P*=.01) in the intervention group but not in the control group (within-group *P*=.85). The extent of improvement in the intervention group was not but almost significantly larger than that in the control group (group × time *P*=.07), as shown in Table 4.

Walking speed, visual short-term memory, and inhibition of cognitive inference did not show more improvement in the intervention group compared to the control group, as shown in Table 4.

### Harms

In all the VRMCT sessions (n=4874) conducted on participants in both intervention and waitlist control groups (including some preintervention trial sessions on some participants), the VR sickness symptoms reported by participants using the VRSQ ranged from 0.7% on symptoms of difficulty focusing and headache to 3% on the symptom of vertigo. Among all the reported VR sickness symptoms, the most reported frequency was “less than half” (ie, 82%-93%). Regarding the most reported VR sickness symptom (ie, vertigo), only participants in 1% of the total sessions reported to have experienced it “more than half” during the VR training. No participants reported severe untoward effects requiring medical treatment after the completion of the training. However, 0.5% of the total training sessions were reported to have technical issues related to the training system.

## Discussion

### Principal Findings

This is one of the very few studies that provides direct evidence using a randomized controlled trial that a VRMCT intervention using a gamified and light-intensity approach is effective in promoting global cognitive function, marginally promoting executive function, and reducing frailty specifically in the at-risk population of older people with cognitive frailty, who also adhered well to the intervention.

Previous studies have shown that motor-cognitive training has positive effects on cognitive function in cognitively healthy older people [48], on executive function in older people [49], and on dual-task walking ability in older people with MCI [50]. It also brings cognitive benefits probably because it yields moderations at the neuronal level (eg, the dopaminergic system showing decreases in mean diffusivity in the frontal and subcortical brain areas in functional magnetic resonance imaging) [51], although the improvement may not be directly due to the moderation of the Aβ metabolism [52]. The findings of this study also agree with previous findings that motor-cognitive training promotes cognitive function and was also effective in a specific group of older people with cognitive frailty [25,49]. This study recommends the use of VRMCT to promote cognitive function and reduce physical frailty for older people with cognitive frailty. However, this study cannot conclude that the improvement in clinical outcomes was caused by or correlated with neuroplastic changes, as proposed in the literature [10]. Future studies should also examine whether this intervention can also yield beneficial moderation at the neuronal level.

The implementation methods and dose are also important. Motor-cognitive training is a general term without a clear gauge of the portion of time spent on simultaneous training throughout the training period. The conventional and effective simultaneous motor-cognitive training protocols usually use 100% motor-cognitive training time throughout the training period [53]. However, recently, some types of gamified motor-cognitive training have adopted a more amusing approach with a lower portion of simultaneous training time and have also demonstrated effectiveness in improving executive function and dual-task performance [24]. The intensity of the training in terms of training hours per session and training period is usually high (eg, 60-minute sessions for 12 weeks), which may not be easily tolerated by the vulnerable group of older people with cognitive frailty [24,48]. This intervention protocol adopted a gamified and light-intensity approach (ie, 30 minutes [actual VRMCT time] per session twice per week for 8 weeks) and also showed effectiveness in promoting cognitive function.

This piece of evidence opens up the possibility of a less serious and less intensive training model implementable in the community to promote the cognitive function of older people. This study recommends that gamified and light-intensity training interventions should be promoted to community-dwelling older people with cognitive frailty in community centers for cognitive promotion purposes. This intervention protocol also adopted training content similar to activities of daily living using VR technology, which aimed at lowering the learning barrier and improving the ecological validity of the training to be translated to improving the daily activity of the participants. We believe that performing these functional tasks with internalized real-time feedback through VR may have a more significant impact on various executive functions, leading to a significantly improved global cognitive function in the experimental group. In addition, the enjoyment and attractiveness of the VR characteristics likely increased motivation and contributed to more extensive training effects on executive function in the experimental group compared to the control group.

Recent evidence has shown that physical frailty prevalence is reduced in older people’s cognitive frailty after the completion of motor-cognitive training [54]. However, the available evidence shows that spontaneous revision to normality from frailty is possible [55]. This study used a more rigorous approach of a randomized controlled trial that showed that the improvement in frailty was significantly greater in the intervention group compared with the control group and where the risk of spontaneous revision to normality was comparable between groups. It provided greater confidence that motor-cognitive training is effective in reducing physical frailty. Although plenty of evidence shows that motor-cognitive training is more effective in promoting physical functions (eg, gait speed, single-task walking function, and balance) than single-task training [11,53], the findings of this study still could not prove whether motor-cognitive training is more effective than single-task training in reducing physical frailty. Future studies should examine whether motor-cognitive training is more effective than single-task training in reducing physical frailty.

The adherence rate of the intervention in this study is also appealing, with 81.6% of participants attending >81% (13/16) of the total number of sessions. A systematic review showed that the average adherence rate to community-based group exercise interventions for older people is only 69.1% [56]. This finding also agrees with the literature on the fact that technology-enhanced (ie, using VR) exercise programs can improve adherence to exercise interventions in older people [57]. Future studies should also compare which technology-enhanced interventions may lead to a better adherence rate.

Despite the rigorous design of this study with favorable outcomes, there are several limitations. First, there were 14.4% (21/146) and 7.5% (11/147) of participants lost to follow-up in the intervention and control groups, respectively. Although the research team executed many measures to reduce the risk of loss to follow-up (eg, sending reminders and providing certificates of completion to those who completed the T1 data collection), it was not avoidable because some centers were closed sporadically in response to the unpredictably varying severity of COVID-19 during the study period. Data collection could not be carried on because some centers were closed for a prolonged time because of COVID-19. Fortunately, the amount of loss to follow-up was only 10.6%, which is <20%, so it is unlikely to pose a serious threat [58]. Second, there was a relatively large group of participants who refused to participate in the Stroop tests (ie, 33/147, 22.4% to 45/146, 30.8%), as shown in Table 3, because they either did not understand the instructions or could not finish the Stroop test within the scheduled period of assessment. Some participants spent too long completing other tests. The Stroop test was arranged as the last task so that it could not be completed by some participants. Therefore, the insignificant results could be caused by unintentional selection bias. Third, this study only evaluated the effects of the intervention on short-term outcomes. Future studies should also evaluate its longer-term effects after the improvement in cognitive function, such as social participation and life-space mobility [31]. Fourth, although a VR element was added to this intervention, because of the trial design (ie, the control group received only usual care), the efficacy of the VR element contributing to favorable clinical outcomes cannot be concluded in this study. Future studies should examine whether VR-integrated motor-cognitive training is more effective than non–VR-integrated motor-cognitive training. Fifth, the generalization of the findings depends on the access to the training prototype. To date, VRMCT is still a prototype, and its effects cannot benefit the target population immediately. Commercialization of the prototype with cost control measures should be implemented to ensure that the effects of VRMCT can be accessible and affordable to benefit the population in an ethical manner, such as regarding equity. Sixth, the contents of VRMCT were all culturally adapted to Hong Kong Chinese culture (eg, language, the virtual environment, and the activities familiar to older people). This culture-specific prototype limits the generalizability and the direct use of VRMCT. Cultural modification is needed to generalize the effects of VRMCT to other cultures and geographic locations. Seventh, only short-term effects of VRMCT (ie, the week immediately after the completion of the intervention) were evaluated. Longer-term effects should be examined in future studies. Eighth, the clinical significance of our results may be minimal given the MoCA increase of only 0.93 points. Therefore, the interpretation of the findings in this study should be approached with caution. In future research, we will explore the impact of the observed MoCA score improvement in the context of daily clinical practice and cognitive function. Specifically, we will assess whether this degree of improvement translates into meaningful benefits for patients, such as enhanced cognitive abilities or improved quality of life. Finally, an unexpected finding showed that the forward digital span scores significantly improved from T0 to T1 in both the intervention and control groups, as shown in Table 4. It might have been caused by the practice effect. Evidence has shown that digit span tests and many commonly used attentional tests do not always have optimal test-retest stability because the practice effect often causes retest scores to be higher [59,60]. Future studies can adopt a composite measure of several tasks or use alternate forms of the measures to yield a more stable result [59,60].

### Conclusions

To conclude, VRMCT is effective in promoting cognitive functions and reducing physical frailty and is also well tolerated and accepted by older people with cognitive frailty, as evidenced by its high attendance rate and negligible VR sickness symptoms. Further studies should examine the efficacy of the intervention components (eg, VR vs non-VR and dual vs single task) on health outcomes, the effect of using technology on intervention adherence, and the long-term effects of the intervention on older people with cognitive frailty at the level of daily living.

## Appendix

Multimedia Appendix 1 Video of the week 2 theme: finding a bus stop.

Multimedia Appendix 2 Video of the week 5 theme: grocery shopping.

Multimedia Appendix 3 The CONSORT (Consolidated Standards of Reporting Trials) checklist.

## Abbreviations

CONSORT: Consolidated Standards of Reporting Trials
FFP: Fried frailty phenotype
MCI: mild cognitive impairment
MoCA: Montreal Cognitive Assessment
TMT: Trail Making Test
VR: virtual reality
VRMCT: virtual reality motor-cognitive training
VRSQ: Virtual Reality Sickness Questionnaire
